# Retrospective Natural History Study of *RPGR*-Related Cone- and Cone-Rod Dystrophies While Expanding the Mutation Spectrum of the Disease

**DOI:** 10.3390/ijms23137189

**Published:** 2022-06-28

**Authors:** Marco Nassisi, Giuseppe De Bartolo, Saddek Mohand-Said, Christel Condroyer, Aline Antonio, Marie-Elise Lancelot, Kinga Bujakowska, Vasily Smirnov, Thomas Pugliese, John Neidhardt, José-Alain Sahel, Christina Zeitz, Isabelle Audo

**Affiliations:** 1 Sorbonne Université, INSERM, CNRS, Institut de la Vision, 75012 Paris, France; marco.nassisi@inserm.fr (M.N.); giusedebartolo@gmail.com (G.D.B.); saddekms@gmail.com (S.M.-S.); christel.condroyer@inserm.fr (C.C.); aline.antonio@inserm.fr (A.A.); marie2074@gmail.com (M.-E.L.); kinga_bujakowska@meei.harvard.edu (K.B.); vasily.smirnov@inserm.fr (V.S.); pugliese.thomas@gmail.com (T.P.); j.sahel@gmail.com (J.-A.S.); 2 Centre Hospitalier National d’Ophtalmologie des Quinze-Vingts, National Rare Disease Center REFERET and INSERM-DGOS CIC 1423, 75012 Paris, France; 3 Department of Clinical Sciences and Community Health, University of Milan, 20122 Milan, Italy; 4 Ophthalmology Unit, Fondazione IRCCS Ca’ Granda Ospedale Maggiore Policlinico di Milano, 20122 Milan, Italy; 5 Ocular Genomics Institute, Massachusetts Eye and Ear Infirmary, Department of Ophthalmology, Harvard Medical School, Boston, MA 02114, USA; 6 Exploration de la Vision et Neuro-Ophthalmologie, Centre Hospitalier Universitaire de Lille, 59000 Lille, France; 7 Faculté de Médecine, Université de Lille, 59000 Lille, France; 8 Human Genetics, Faculty VI, School of Medicine and Health Sciences, University of Oldenburg, 26129 Oldenburg, Germany; john.neidhardt@uni-oldenburg.de; 9 Research Center Neurosensory Science, University Oldenburg, 26129 Oldenburg, Germany; 10 Department of Ophthalmology, University of Pittsburgh Medical School, Pittsburgh, PA 15213, USA

**Keywords:** genotype–phenotype correlation, *RPGR*, *RPGR*-related retinal dystrophies, *RPGR*-related cone dystrophy, *RPGR*-related cone-rod dystrophy

## Abstract

Variants in the X-linked retinitis pigmentosa GTPase regulator gene (*RPGR)* and, specifically, in its retinal opening reading frame-15 isoform (*RPGR^ORF15^*) may cause rod-cone (RCD), cone, and cone-rod dystrophies (CDs and CRDs). While *RPGR*-related RCDs have been frequently evaluated, the characteristics and progression of *RPGR*-related CD/CRDs are largely unknown. Therefore, the goal of our work was to perform genotype–phenotype correlations specifically in *RPGR^ORF15^*-related CD/CRDs. This retrospective longitudinal study included 34 index patients and two affected relatives with a molecular diagnosis of *RPGR*-related CD/CRDs. Patients were recruited at the “Quinze-Vingts” Hospital, Paris, France and screened for mutations in *RPGR^ORF15^* at the Institut de la Vision, Paris, France. We identified 29 distinct variants, of which 27 were truncating. All were located in the 3′ half of the *RPGR^ORF15^* transcript. Twenty of them were novel. Fifteen subjects were affected by CD, the remaining had CRD. When analyzing the longitudinal data, a progressive decline in visual acuity (VA) was noted, with more than 60% of the patients reaching VA ≥ 1 LogMar in the best eye after the fifth decade of life. To our knowledge, this is the largest described study of a cohort of CD/CRD patients affected by *RPGR^ORF15^* variants. Longitudinal data showed a rapidly progressive disease, possibly locating an optimal window of intervention for future therapies in younger ages.

## 1. Introduction

Cone/cone-rod dystrophies (CDs and CRDs) are inherited retinal progressive diseases that primarily involve cone degeneration. Occasionally, it may be followed by rod degeneration [1]. Patients usually experience photophobia, color vision abnormalities, and the loss of central vision from the very early stages of the disease [1]. More than 30 genes and every monogenic mode of inheritance have been associated with these dystrophies (data from https://sph.uth.edu/retnet/, last access: 1 January 2022). A common cause of X-linked CD or CRD are variants in the retinitis pigmentosa GTPase regulator gene (*RPGR,* MIM# 312610) [2]. This gene has been associated with several disease patterns including rod-cone dystrophy (RCD, 70%), CRD (6–23%), and CD (7%) [3,4]. Three major *RPGR* isoforms are expressed in the human retina: (i) an isoform including exons 1 to 19 (*RPGR*^ex1−19^); (ii) an isoform skipping exons 14 and 15; and (iii) an opening reading frame-15 isoform (*RPGR^ORF15^*) [5,6,7]. The *RPGR^ORF15^* shares the first 15 exons with *RPGR*^ex1−19^, then parts of intron 15 are included into the transcript to generate the exon *ORF15* [8]. The *ORF15* exon encodes for a highly repetitive domain and is considered as a mutational “hot spot”, with most disease-associated variants being truncating [8]. *RPGR^ORF15^* is strongly expressed in the connecting cilium of photoreceptors where it is implicated in scaffolding, protein trafficking, and quality checking between the inner and outer segments [5,9,10,11,12]. Interestingly, CD and CRD are predominantly associated with variants toward the 3′ end of the *ORF15* exon in *RPGR^ORF15^*, with a “watershed zone” (approximately residues 949–1047 residue) where variants have been associated with both CD/CRD and RCD [13,14]. While *RPGR*-related RCDs have frequently been evaluated clinically, the characteristics and progression of *RPGR*-related CD/CRDs are not well-understood, with very few reports available in the literature that have studied relatively small cohorts [4,15,16]. In the era of gene therapy, a continuous improvement in the knowledge on the natural disease courses is required to select patients who are most likely to benefit from gene therapy and to identify an optimal therapeutic window of intervention. In this study, we identified a cohort of 36 patients with *RPGR^ORF15^-*related CD/CRDs. We analyzed several clinical parameters and performed a genotype–phenotype correlation.

## 2. Results

### 2.1. Genetic Screening

**Figure 1:**
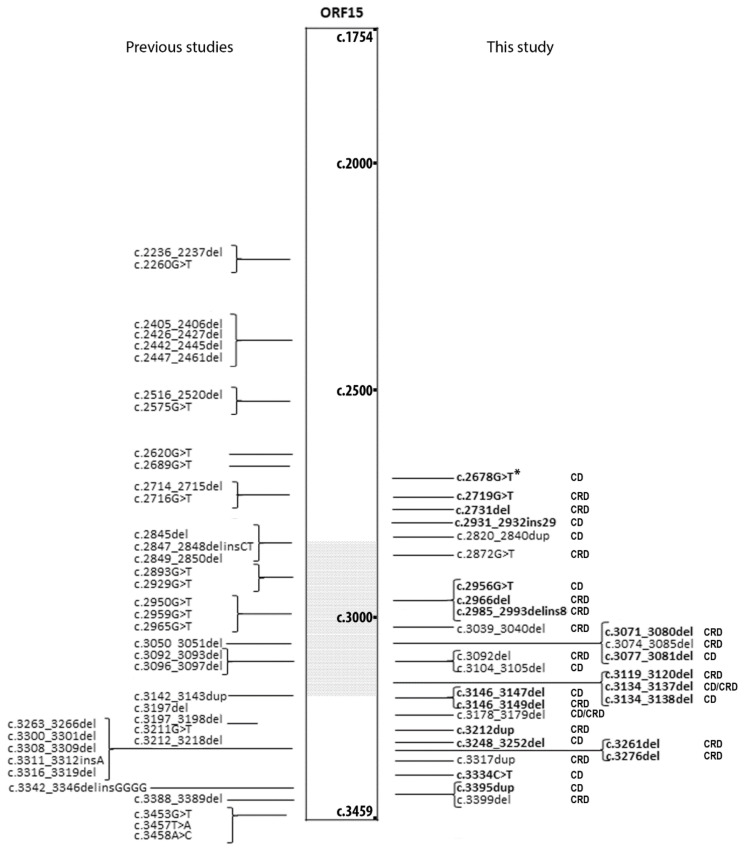
The list of *RPGR^ORF15^* (NM_001034853.2) variants associated with cone or cone-rod dystrophies (CDs or CRDs). Variants that have been previously published by other groups are depicted on the left. Variants reported in the present study are on the right with the associated phenotype. Novel variants are reported in bold. The grey dotted area corresponds to the “watershed zone” as approximately defined by De Silva et al. ^10^. *: Variant c.2678G>T is a variant of unknown significance.

A total number of 34 index subjects (and two affected relatives) recruited at the Centre of Rare Diseases at Quinze-Vingts Hospital were diagnosed with *RPGR^ORF15^*-related CD/CRDs after genetic screening (Table 1, Figure S1). Overall, we identified 29 distinct variants, of which 27 (93.1%) were frameshift or nonsense variants (Figure 1). Twenty-four variants (82.8%) were localized between residue 949 and the C-terminus of the protein, with 13 of them in the “watershed zone”, as defined by De Silva et al. (i.e., between residues 949 and 1047 [13,14]; Figure 1). Among all of the variants, 20 were novel: 12 small deletions, two small duplications, one small deletion-insertion, one insertion, one missense, and three nonsense mutations (Figure 1 and Table 2). According to the criteria of the American College of Medical Genetics and Genomics (ACMG) [17], 19 of the 20 novel variants were pathogenic or likely pathogenic, while one was of uncertain significance (Table 2). The latter, c.2678G>T p.(Gly893Val), was predicted to be pathogenic by two in silico algorithms (Table S1), affecting a moderately/low conserved position (Table S2).

### 2.2. Clinical Cross-Sectional Data

For the phenotype analysis, data from all of the affected subjects were included and analyzed (34 index patients and two affected relatives). According to the ff-electroretinograms (ERGs) in their last visit available, the phenotype of 15 subjects was classified as CD, while the rest showed functional signs of both cone and rod degeneration (CRD). The onset of the disease was in the first two decades for 72% of patients (13 out of 18 with available data). All clinical and functional data are summarized in Table 3 and are reported in detail in Table S3. The mean age at the last examination was 43.97 ± 11.24 years (range: 9–73 years) and the mean best corrected visual acuity (BCVA) was 0.97 ± 0.71 LogMAR, with only one patient having light perception in at least one eye. Color vision was altered in most patients (96.87%), however, no clear preference toward a definite color axis was evident.

**Figure 2:**
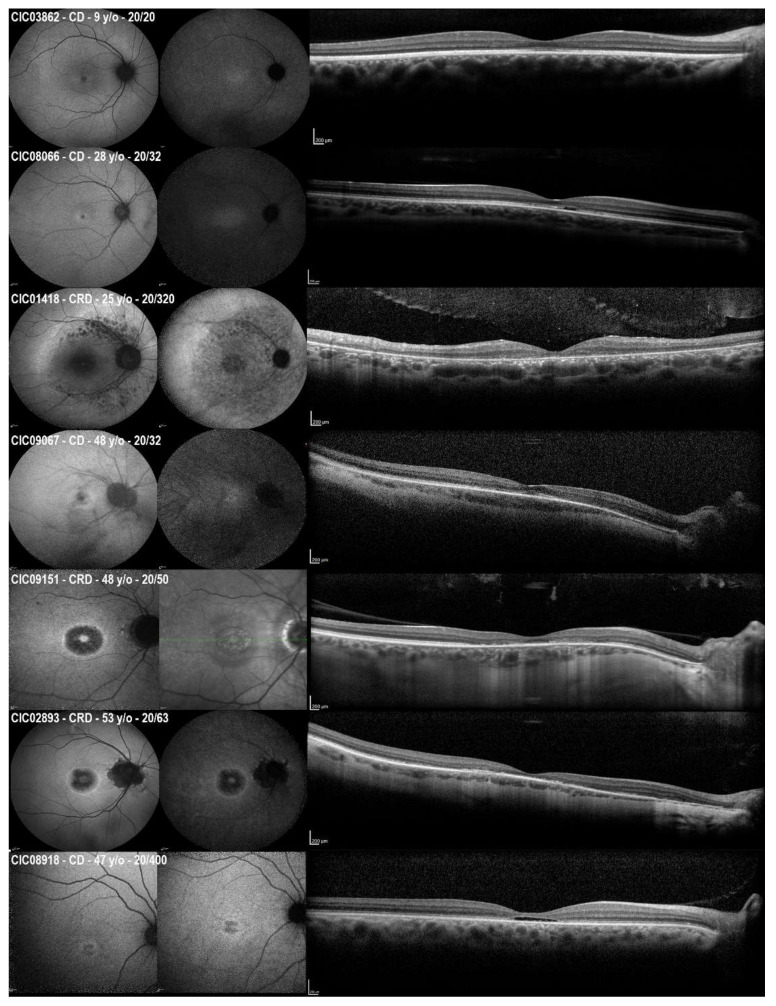
The short-wavelength fundus autofluorescence (SW-FAF, **left**), near-infrared fundus autofluorescence (NIR-FAF, middle), and optical coherence tomography (OCT, **right**) of six patients with CD/CRD included in the study. For CIC09151, the NIR-FAF was not available and was replaced with a near-infrared reflectance image. Phenotypes may range from the focal central alterations (e.g., in CIC03862 CIC08066, CIC09067, CIC08918) to a progressively more extended disease (e.g., CIC09151, CIC02893, and CIC01418). Patients with similar ages may show different degrees of retinal degenerations. CIC09067 and CIC09151 are affected brothers that showed different phenotypes (CD and CRD, respectively) with different retinal involvement, despite sharing the same *RPGR^ORF15^* pathogenic variant and having the same age at the time of examination.

The qualitative analysis of short-wavelength fundus autofluorescence (SW-FAF) revealed that most subjects belonged to group 2 (60%) with the area of central hypoautofluorescence greater than one disk diameter (DD) but within the macula. This area represents the central outer retinal layer atrophy evident in the spectral domain optical coherence tomography (SD-OCT) (Figure 2). Three subjects showed foveal lacunae (e.g., CIC08918 in Figure 2). In very mild or early cases, retinal alterations started with an altered reflectance and/or the interruption of the foveal ellipsoid zone (EZ) line on SD-OCT, which corresponded to a central hyperautofluorescence on the SW-FAF due to the window defect (Figure 2). When comparing the age of the three SW-FAF groups, patients in group 1 were younger (38 ± 14.74 years) than in group 2 (45.62 ± 9.49 years) and group 3 (45.71 ± 12.64), but this difference was not statistically significant (independent samples Kruskal–Wallis test, *p* = 0.662).

### 2.3. Longitudinal Data on SW-FAF

**Figure 3:**
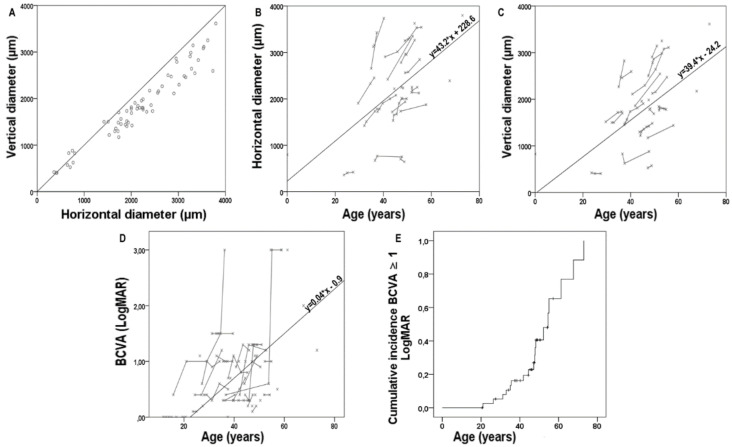
The scatterplots of all the horizontal and vertical diameters (**A**) of the central hyperautofluorescent (hyperAF) ring as seen on the short-wavelength fundus autofluorescence, plotted together. The black line is the reference line. (**B**,**C**) are the scatterplots of the same horizontal (**B**) and vertical (**C**) measurements grouped per patient and plotted with age. (**D**) The scatterplot of all best corrected visual acuity (BCVA) measurements available plotted with age. Each grey line represents the measurements from a single subject. In (**B**–**D**), the black line is the function of the mean regression slope and intercept derived from the individual regression slopes and intercepts of patients with the available longitudinal data. (**E**) Kaplan–Meier survival curves showing the cumulative incidence of BCVA ≥ 1 LogMar in the best eye for *RPGR^ORF15^*-related cone and cone-rod dystrophies in patients as a function of age.

A total number of 27 patients (75%) showed a hyperAF ring surrounding the central dystrophic area. For 21 patients, we had multiple available visits, thus allowing for the estimation of the enlargement of its diameters. Two patients were excluded from these measurements as the hyperAF ring either included the optic nerve head or disappeared during the follow-up (Figure S2). A total of 54 measurements from 19 patients were collected. At the baseline, the horizontal and vertical diameters were 1948.2 ± 810.6 and 1619.2 ± 648.9 µm (*p* < 0.001), respectively. In contrast, at the last visit available to the study, these were 2216.7 ± 962.5 and 1853.7 ± 791.1 µm (*p* < 0.001). As shown in Figure 2, the central hypoAF area tended to adopt a circular shape for smaller diameters (<1000 µm) and an ellipsoid shape for larger diameters. All measurements were plotted with age to estimate the rate of enlargement of the hyperAF ring diameters using individual regression slopes; the mean regression slope was 43.16 ± 47.81 and 39.36 ± 40.57 for the horizontal and the vertical diameters, respectively (Figure 3).

### 2.4. Longitudinal Data on Visual Acuity and Correlations

A total number of 98 BCVA measurements were collected from all available visits from the overall cohort. All of these measurements were plotted with age to estimate the rate of BCVA decline using individual regression slopes (Figure 3); the mean regression slope was 0.04 ± 0.08 and the mean intercept was −0.90 ± 3.34 (Figure 3). Furthermore, a Kaplan–Meier survival curve was built to show the cumulative incidence of BCVA ≥ 1 LogMar (20/200 Snellen) in the best eye as a function of age (Figure 3). This incidence reached 60% of the CRD patients before 60 years of age. An attempt of correlation between the visual acuity and all of the other parameters was conducted using a linear regression analysis with the cross-sectional data from the last visit with a stepwise approach (Table 4). The most important predictors of BCVA seem to be related to the imaging, with a significant correlation found for the central retinal thickness (CRT; β coeff. −0.523, *p* = 0.003), the SW-FAF phenotype (β coeff. 0.353, *p* = 0.044), and the peripapillary sparing (β coeff. −0.376, *p* = 0.031). The model improved when both the CRT and peripapillary sparing were considered together, with an overall coefficient R = 0.666.

### 2.5. Genotype–Phenotype Correlation

Given the relatively small cohort, it was impossible to draw definitive conclusions on the genotype–phenotype correlation. The only missense variant found in the cohort (i.e., c.2678G>T p.(Gly893Val), in CIC08918) was associated with CD, foveal focal loss of the EZ line, and low BCVA (20/400; Figure 2). Therefore, the location of the variants along the *ORF15* does not seem to affect the development of either the CD or CRD, with some variants that could lead to both, even in related subjects (i.e., c.3178_3179del p.(Glu1060Argfs*18) in CIC09067 and CIC09161; Figure 2).

## 3. Discussion

**Figure 4:**
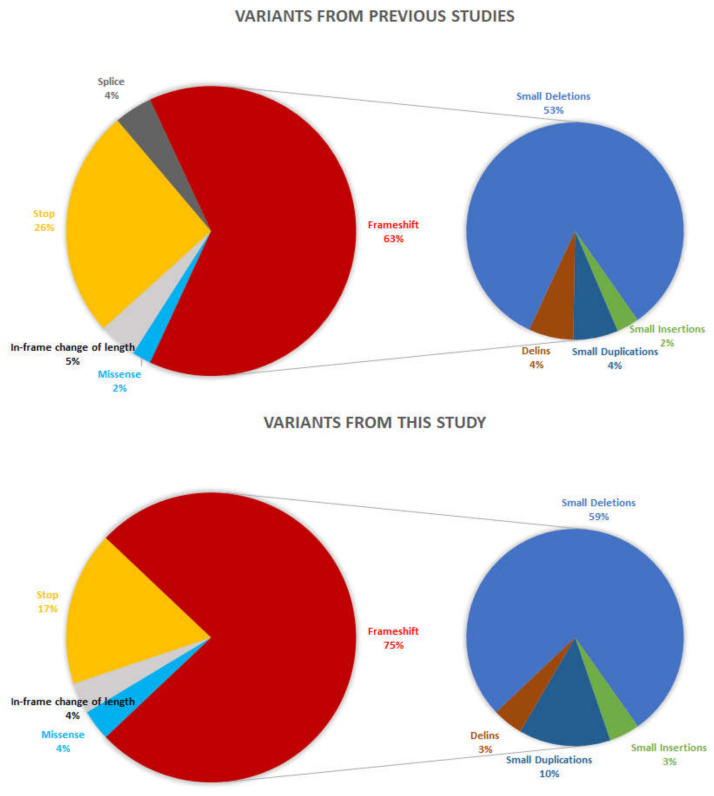
Pie charts showing the distribution of the *RPGR^ORF15^* variants underlying cone/cone-rod dystrophies according to their types. The distribution of the variants that have previously been published by other groups is depicted at the top. Variants reported in the present study are on the bottom.

X-linked inheritance is uncommon in patients with CD or CRD (around 1%) [25]; even so, variants in *RPGR* are responsible for 73% of them [26]. *RPGR-*related CD/CRD has been associated only with variants in the *RPGR^ORF15^* isoform, which, however, may also be associated with RCD. Until now, among the 277 described *RPGR^ORF15^* inherited retinal disease variants (data from HGMDPro database [27], last access on 1 January 2022), only 47 were associated with CD/CRD. Our study broadens the mutation spectrum of *RPGR^ORF15^* related CD/CRD with 20 novel variants, two of which were predicted to be pathogenic and 17 were predicted to be likely pathogenic, according to the ACMG criteria. As expected, 92% of variants were frameshift or nonsense (93% in a previous study, Figure 4). Most of the variants were identified within a single family. The latter might be related to the relatively poor representation of *RPGR*-related CD/CRD cohorts in the literature, together with the rarity of this condition. Indeed, *ORF15* sequencing relies on traditional Sanger sequencing because of the technical difficulties secondary to the high repeatability of this domain; hence, few laboratories are able to screen for it [28,29,30,31]. However, in the near future, more data should be available since novel next generation sequencing methods will better cover this region [32].

Furthermore, they were located toward the 3′ end of the ORF15 region of *RPGR* previously associated with CD/CRD and in the “watershed zone” between the 949th and the 1047th amino acid residues, previously associated with either CD/CRD or RCD [14]. The reason behind this variability is yet to be discovered, but it can be speculated that alterations in protein–protein interactions influence the outcome and progression of the disease [12,33]. *RPGR^ORF15^* localizes at the photoreceptor cilium with a key role in ciliary function and protein trafficking [5]. *RPGR^ORF15^* interacts with several proteins including RPGRIP1, PDE6D, TTLL5, and *CEP290* [12,33,34,35] and these interactions may vary between the rods and cones, even though the underlying mechanisms are still unknown. However, so far, no specific protein interactions have been identified with the protein part encoded by the *RPGR* exon ORF15, whose domain is predicted to be unstructured. One hypothesis is that variants close to the 3′-end may not lead to mRNA nonsense-mediated decay, hence, the resulting truncated proteins may worsen the loss-of-function through a potential gain-of-function or dominant negative mechanism [36,37]. On the other hand, other photoreceptor-specific proteins containing glutamic acid-rich domains (i.e., CNGB1) have been associated with retinal dystrophies, even though their interactions have been better defined [38,39,40]. The boundaries of the “watershed zone” may not be precise (Figure 1). Indeed, 13 variants from previous studies and the four novel variants described herein are more proximal than this area, indicating that the interaction of RPGR in the different cell types may be more complex and/or the influence of other genetic modifiers and environmental factors on the phenotype may be more relevant than expected. Indeed, the effect of oxidative stress and pro-inflammatory cytokines induced by environmental factors (e.g., light) have been suggested to accelerate retinal degeneration through photoreceptor and retinal pigment epithelium damage [41,42,43]. Further studies will be needed to test these hypotheses.

Few studies have focused on *RPGR*-related CD/CRD describing the disease phenotype and progression [4,15,16]. To the best of our knowledge, this is the largest cohort reported thus far. As expected from a CD/CRD phenotype, visual acuity tends to deteriorate faster than in patients with RCD. All included patients showed ERG patterns with diminished or undetectable photopic responses, while scotopic responses could be either normal (CD) or reduced/absent (CRD). Unfortunately, we did not find any correlation between the variants and the presence of scotopic alterations. An association between RPGR-related dystrophies and the presence of high myopia [4] has already been reported, however, the molecular mechanisms behind this are still unknown. With regard to glaucoma (two patients) and retinal detachment (one patient), it was impossible to draw definitive conclusions on the genotype–phenotype correlation and given their small prevalence, their findings may be coincidental.

In our cohort, we found a rate of BCVA decline of about 7%/year (two ETDRS letters per year), with most of the patients reaching a BCVA ≥ 1 LogMar (≤20/200 Snellen equivalent) during the fifth decade of life. These data are coherent with the previous literature [4,15] and help to locate a hypothetical window of therapeutic intervention for these patients as early as possible. SW-FAF was mostly characterized by a central area of hypoAF representing the dystrophic/atrophic retinal tissue, surrounded by a ring of hyperAF (27 patients, 77.1%), which increases in size with the advance of the disease [4,25]. This increase is not morphologically symmetric: initially, the hyperAF ring is circular, then the horizontal diameter becomes wider, giving the ring an ellipsoid shape. Interestingly, this resembles our findings toward the progression of the hyperAF ring in RCD cohorts [44,45]. In RCDs, the hyperAF ring at the posterior pole comprises an area of preserved retina, hence, its significance is the opposite to that of the CD/CRD ring [46]. The hyperAF ring tends to shrink with RCD progression, starting from an ellipsoid shape at higher diameters to a circular shape at smaller ones [44,45]. This is similar, but reversed, to what happens in CD/CRD. These observations suggest a non-random pattern of photoreceptor degeneration: specifically, a “higher resistance” to damage may be present on the vertical diameter (slower rate of shrinking in RCD and slower rate of widening in CD/CRD) than on the horizontal diameter. This phenomenon might be related to the topographic distribution of photoreceptors in the retina, which is characterized by a higher density of cones on the horizontal meridian that degenerates faster in the early stages of CD/CRD and in the late stages of RCD [47]. On the other hand, the horizontal raphe is the demarcation line between the two vascular hemispheres of the retina and the choroid [48], hence it might be less efficient in the metabolization of the cellular debris generated by the photoreceptor degeneration, increasing their toxic effect on residual cells.

In this study, we tested whether BCVA could be predicted by several parameters described in the CD/CRD patients associated with *RPGR* variants. Indeed, three imaging parameters demonstrated a good association with BCVA and two of them (i.e., peripapillary sparing and CRT) reached an overall correlation coefficient of 0.666. Peripapillary sparing is a feature that can be present in different inherited retinal dystrophies and its prognostic value has already been demonstrated in Stargardt disease [49,50,51,52]. It is not clear whether this specific sign is due to a more favorable photoreceptor/retinal pigment epithelium ratio in the peripapillary area, and/or to the presence of a thicker retinal nerve fiber layer that better shields this area from oxidative light damage [53]. The high prevalence of high myopia (42% in our cohort and 50 to 72% in previous studies [4,15]) in *RPGR*-related CD/CRD may influence the presence of this feature. Of note, peripapillary involvement appears more circular when it is myopia-associated and more irregular when occurring without myopia; however, regardless of its origin and pathogenicity, peripapillary sparing may be an important prognostic factor in *RPGR*-related CD/CRD patients. Future prospective longitudinal studies are warranted to confirm these data.

Our study had several limitations including the retrospective design and the relatively small sample size, which, however, is related to the rarity of this condition. Nevertheless, the study has also strengths such as the presence of a molecular diagnosis for all subjects and the standardization of all investigations within the same tertiary referral center.

## 4. Materials and Methods

All patients were clinically evaluated at the “National Reference Center for Rare Retinal Diseases” of the “Quinze-Vingts” Hospital, Paris, France. Clinical charts and all exams were retrospectively reviewed and analyzed. Only patients with a molecular diagnosis of *RPGR*-related CD/CRD were included in the study.

### 4.1. Mutation Analysis

Mutation analysis of *RPGR^ORF15^* variants and cosegregation analyses on available family members were performed using Sanger sequencing, similarly to that previously described at the “Institut de la Vision”, Paris, France [54]. All patients donated a peripheral blood sample and total genomic DNA was extracted according to the manufacturer’s recommendation (Puregen Kit; Qiagen, Courtaboeuf, France). Patients with a cone or cone-rod dystrophy for which an X-linked pattern was suspected were investigated for *RPGR^ORF15^* variants through Sanger sequencing. The *RPGR^ORF15^* exon 15 and its flanking intronic regions were amplified in a single fragment (*RPGR^ORF15^* RefSeq NM_001034853.2) using oligonucleotides reported in Table S4, a commercially available polymerase (HotFire, Solis Biodyne, Tartu, Estonia), and 3 mM MgCl_2_ at an annealing temperature of 60 °C for 1 min. Polymerase chain reaction (PCR) products were enzymatically purified (ExoSAP-IT, USB Corporation, Cleveland, Ohio, USA purchased from GE Healthcare, Orsay, France): 5 μL of ExoSAP-IT (1/50) was added to 0.2 μL of the PCR product for 15 min at 37 °C to activate the enzyme and then the temperature was increased to 80 °C for 15 min to inactivate the enzyme. After the enzymatic purification step, a sequencing-specific PCR was performed using specific oligonucleotides (Table S4). The mixes were subjected to 30 cycles of PCR. Each cycle was composed of a denaturation step (20 s at 96 °C), a hybridization step (10 s at 56 °C for ORF15_R4Seq, ORF15_R5Seq, ORF15_R9Seq, and ORF15_R11Seq; 58 °C for ORF15_R7bSeq; 63° for ORF15_R8bSeq) and an extension step (4 min at 60 °C for ORF15_R4Seq, ORF15_R5Seq, ORF15_R9Seq, and ORF15_R11Seq; 63 °C for ORF15_R7bSeq; 68° for ORF15_R8bSeq). After this PCR, a final cleaning step of the PCR product was necessary before DNA sequencing. This purification was performed in 96-well plates containing filters of 45 μm pores, each of which was filled with Sephadex G-50 powder (GE Healthcare, Orsay, France). The plates were hydrated for 3 h at room temperature with 300 μL of sterile water. Subsequently, centrifugation for 1 min at 15,000× *g* was performed. Then, in each well, 150 μL of sterile water was added. The plates were centrifuged for 5 min at 950× *g*. Finally, in each well, 15 μL of sterile water and 10 μL of the sequence reaction were added. A final centrifugation was performed for 5 min at 910× *g*. The eluate was recovered and analyzed by a 48-capillary sequencer (3730 DNA Analyzer, Applied Biosystems, Waltham, MA, USA). Sequences of the patients were compared to the reference *RPGR^ORF15^* sequence (GRCh38, NM_001034853.2) using sequencing software (SeqScape Software v.2.6, Applied Biosystems). All variants were classified following the American College of Medical Genetics and Genomics (ACMG) guidelines and standards [17] and the Association of Molecular Pathology (AMP) Clinical Practice Guidelines and Reports based on previous publications (as compiled in the Human Gene Mutation Database (HGMD) [27], and in the Leiden Open (source) Variation Database (LOVD) [55], population data, computational data, and functional data. The Genome Aggregation database (gnomAD, available at https://gnomad.broadinstitute.org/) was used to check the variant frequency. Novel variants with unknown frequency or minor allele frequency (MAF) ≤ 0.05 were further tested using Alamut Visual software v. 2.7.1, where the in silico predictive programs PolyPhen2 (Polymorphism Phenotyping, http://genetics.bwh.harvard.edu/pph2/) [56], SIFT (Sorting Intolerant from Tolerant; http://sift.bii.a-star.edu.sg/) [57], and Mutation Taster (http://www.mutationtaster.org/) [58] were implemented. Evolutionary conservation was investigated using the 46-way Vertebrate Multiz Alignment and Conservation of the University of California Santa Cruz (UCSC) genome browser [59,60]. For missense mutations, an amino acid residue was considered highly conserved if the same residue was present in all species or was different in just one species among fishes or reptiles; moderately conserved if different in two to five species (included); and not conserved if different in more than five species or in at least one primate. A novel sequence variant was considered pathogenic if it represented a nonsense variant or small insertion, deletion, or duplication, inducing a frame-shift. In the case of a missense change, a novel sequence variant was considered to be likely pathogenic if it was either predicted to be deleterious by all three prediction algorithms or if it affected a highly or moderately evolutionary conserved amino acid residue and was predicted to be pathogenic by at least one algorithm above-mentioned [17]. In all other cases, a variant was classified as a VUS. The same criteria described above, but considering the DNA sequence, were applied to study the conservation of nucleotide residues and the pathogenicity of variants on putative or non-canonic splice sites (±10 bases from exon boundaries).

### 4.2. Clinical Data Collection

Medical records were examined retrospectively to collect the following data: sex, age at onset of symptoms, ophthalmic history (cataract and/or cataract surgery, high myopia), BCVA (i.e., the best possible vision that an eye can achieve with the use of glasses or contact lenses) using the Early Treatment Diabetic Retinopathy Study (ETDRS) charts, slit lamp examination, color vision tested by the desaturated Farnsworth Panel D-15, Goldmann kinetic visual fields (VFs), full-field electroretinography (ff-ERG; Espion E2; Diagnosys, Lowell, MA, USA) performed according to the standards of the International Society for Clinical Electrophysiology of Vision [61], SD-OCT (Spectralis Heidelberg Retina Angiograph [HRA]+OCT; Heidelberg Engineering, Dossenheim, Germany), retinal fundus photography, near infrared fundus autofluorescence (NIR-FAF; HRA II; Heidelberg Engineering, Dossenheim, Germany), and SW-FAF (HRA II; Heidelberg Engineering, Dossenheim, Germany) imaging. The distinction between CD and CRD was based on the presence of scotopic dysfunction on ff-ERG for the latter. For patients with alterations in both the scotopic and photopic responses, we collected the amplitude of the b-wave on the 0.01 dark-adapted ERG (scotopic) and 3.0 light-adapted ERG (photopic). We then calculated the percentage of the mean amplitudes from a cohort of healthy subjects. Patients whose scotopic dysfunction was more severe (defined as a lower percentage of the mean) were considered to have CRD. For patients with undetectable responses at the last visit, ff-ERGs with detectable traces from previous visits were used for the classification. In the case of no previous visits, the classification was based on the visual field and retinal imaging (SD-OCT and fundus autofluorescence) phenotype.

**Figure 5:**
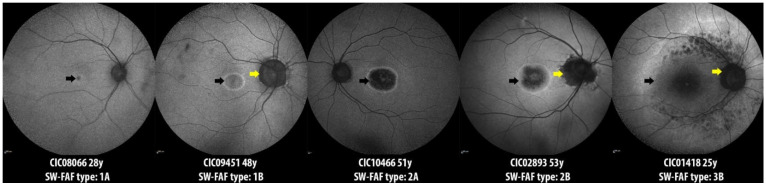
The short wavelength fundus autofluorescence (SW-FAF) qualitative analysis. Patients were classified according to the SW-FAF pattern as follows: group 1—the dimension of the central hypoautofluorescence (black arrows) is inferior to 1 disc diameter (DD); group 2—the dimension of the central hypoautofluorescence exceeds a 1 disc diameter (DD) but stays within the macula; group 3—the central hypoautofluorescence extends beyond the macula. Furthermore, two subgroups were considered: subgroup A when peripapillary sparing was present; subgroup B when the peripapillary area was involved by the disease (yellow arrows).

A qualitative assessment of the SW-FAF was performed according to the following criteria: group 1—the dimension of the central hypoautofluorescence (hypoAF) is inferior to 1 DD; group 2—the dimension of the central hypoAF is superior to 1 DD, but stays within the macula; group 3—the central hypoAF extended beyond the macula (Figure 5). Furthermore, the involvement of the peripapillary area was also considered when present (see Figure 5). A quantitative assessment was also performed on SW-FAF by measuring the horizontal and vertical diameters of the inner border of the hyperautofluorescent (hyperAF) ring encircling the central atrophic area. On the SD-OCT scans, the presence of intraretinal hyper-reflective foci (iHRF) and epiretinal membrane (ERM) were also recorded. The CRT was automatically collected using the machine’s software (Heidelberg Eye Explorer, version 1.9.10.0, Heidelberg Engineering).

### 4.3. Statistical Analysis

Data analysis was performed using IBM SPSS Statistics software v. 21.0 (Chicago, IL, USA). First, the agreement between eyes was tested for BCVA and CRT; since there were no significant differences (see Table S5), all further analyses were performed using the data from the right eye. A stepwise linear regression analysis investigating the association between BCVA and all clinical parameters was also performed for each group and for the entire cohort using cross-sectional data from the last visit available. Finally, longitudinal data from all patients were used to build Kaplan–Meier survival curves showing the survival distribution of BCVA ≥ 1 LogMar (20/200 Snellen) in the best eye from the first visit to our center. For all statistical tests, a *p* value inferior to 0.05 was considered statistically significant.

## 5. Conclusions

Our study broadened the mutation spectrum of *RPGR*-related CD/CRD, confirmed its fast progression, and demonstrated the association of visual acuity with several imaging biomarkers. Overall, this comprehensive analysis will constitute an important guidance in the design of therapeutic clinical trials and will help clinicians in assessing the visual prognosis in patients with *RPGR*-related CD/CRD.

## Figures and Tables

**Table 1:** The distinct variants in index patients with RPGR-related cone-rod dystrophy and cosegregation analysis. Nucleotide positions correspond to NM_001034853.2 for *RPGR^ORF15^*.

ID	Sex		cDNA	Protein Change	Reference
CIC03862	M	Index	c.2931_2932ins29	p.(Glu978Lysfs*121)	This study
782819	M	Index	c.3276del	p.(Gly1093Aspfs*3)	This study
CIC09352	M	Index	c.3261del	p.(Val1088*)	This study
CIC04404	M	Index	c.3178_3179del	p.(Glu1060Argfs*18)	García-Hoyos, 2006 [18]
CIC02893	M	Index	c.3399del	p.(Pro1134Hisfs*18)	Mawatary, 2019 [19]
CIC03560	M	Index	c.3248_3252del	p.(Glu1083Valfs*17)	This study
CIC06538	M	Index	c.3317dup	p.(Ser1107Valfs*4)	Demirci, 2005 [20]
CIC02863	M	Index	c.2719G>T	p.(Glu907*)	This study
CIC07494	M	Index	c.3039_3040del	p.(Glu1014Glyfs*64)	Zahid, 2013 [16]
CIC06631	M	Index	c.3119_3120del	p.(Glu1040Glyfs*38)	This study
			c.3074_3085del	p.(Val1025_Glu1028del)	Olm, 2019 [21]
CIC04447	M	Index	c.3134_3137del	p.(Glu1045Glyfs*43)	This study
CIC04647	M	Index	c.2872G>T	p.(Glu958*)	Shu, 2007 [22]
CIC09451	M	Index	c.3178_3179del	p.(Glu1060Argfs*18)	García-Hoyos, 2006 [18]
CIC07574	M	Index	c.3039_3040del	p.(Glu1014Glyfs*64)	Zahid, 2013 [16]
CIC07658	M	Index	c.2985_2993delinsAGAAGGGG	p.(Glu997Glyfs*92)	This study
CIC07798	M	Index	c.3146_3147del	p.(Glu1049Glyfs*29)	This study
CIC08152	M	Index	c.3334C>T	p.(Gln1112*)	This study
CIC08901	M	Index	c.3212dup	p.(Thr1072Aspfs*7)	This study
CIC09159	M	Affected brother	c.3212dup	p.(Thr1072Aspfs*7)	This study
CIC08918	M	Index	c.2678G>T	p.(Gly893Val)	This study
CIC09067	M	Index	c.3178_3179del	p.(Glu1060Argfs*18)	García-Hoyos, 2006 [18]
CIC09151	M	Affected brother	c.3178_3179del	p.(Glu1060Argfs*18)	García-Hoyos, 2006 [18]
CIC8950	M	Index	c.3104_3105del	p.(Glu1035Glyfs*43)	Mawatary, 2019 [19]
CIC01063	M	Index	c.3074_3085del	p.(Val1025_Glu1028del)	Olm, 2019 [21]
			c.3092del	p.(Glu1031Glyfs*58)	Bader, 2003 [23]
CIC10466	M	Index	c.3395dup	p.(Asn1132Lysfs*12)	This study
CIC09949	M	Index	c.2966del	p.(Glu989Glyfs*100)	This study
CIC10733	M	Index	c.3134_3137del	p.(Glu1045Glyfs*43)	This study
CIC08066	M	Index	c.3178_3179del	p.(Glu1060Argfs*18)	García-Hoyos, 2006 [18]
CIC04835	M	Index	c.2956G>T	p.(Gly986*)	This study
CIC03515	M	Index	c.3178_3179del	p.(Glu1060Argfs*18)	García-Hoyos, 2006 [18]
CIC07400	M	Index	c.3077_3081del	p.(Glu1026Glyfs*51)	This study
CIC09653	M	Index	c.2236_2237del	p.(Glu746Argfs*23)	Vervoort, 2000 [8]
CIC04850	M	Index	c.3071_3080del	p.(Glu1024Glyfs*62)	This study
CIC01503	M	Index	c.2820_2840dup	p.(Asp943_Glu949dup)	Vervoort, 2000 [8]
			c.3134_3138del	p.(Glu1045Glyfs*32)	This study
1659193	M	Index	c.2731G>T	p.(Glu911*)	This study
CIC01418	M	Index	c.3146_3149del	p.(Glu1049Glyfs*39)	This study

**Table 2:** A list of novel hemizygous *RPGR^ORF15^* variants detected in the study.

Genomic Start Position (hg19)	cDNA *RPGR^ORF15^*: NM_001034853.2	Protein Change	ACMG Classification (Criteria)
chrX-38145574	c.2678G>T	p.(Gly893Val)	Uncertain significance (PM2, PP3, BP1)
chrX-38145533	c.2719G>T	p.(Glu907*)	Likely Pathogenic (PVS1, PM2)
chrX-38145521	c.2731del	p.(Glu911Argfs*178)	Pathogenic (PVS1, PM2, PP1)
chrX-38145321	c.2931_2932ins AAGGAAAAGGGGAGAAGGGGAAGGGGAGGAAGGA	p.(Glu978Lysfs*121)	Likely Pathogenic (PVS1, PM2)
chrX-38145296	c.2956G>T	p.(Gly986*)	Likely Pathogenic (PVS1, PM2)
chrX-38145286	c.2966del	p.(Glu989Glyfs*100)	Likely Pathogenic (PVS1, PM2)
chrX-38145259	c.2985_2993delinsAGAAGGGG	p.(Glu997Glyfs*92)	Likely Pathogenic (PVS1, PM2)
chrX-38145172	c.3071_3080del	p.(Glu1024Glyfs*62)	Likely Pathogenic (PVS1, PM2)
chrX-38145171	c.3077_3081del	p.(Glu1026Glyfs*51)	Likely Pathogenic (PVS1, PM2)
chrX-38145132	c.3119_3120del	p.(Glu1040Glyfs*38)	Likely Pathogenic (PVS1, PM2)
chrX-38145115	c.3134_3137del	p.(Glu1045Glyfs*43)	Likely Pathogenic (PVS1, PM2)
chrX-38145115	c.3134_3138del	p.(Glu1045Glyfs*32)	Likely Pathogenic (PVS1, PM2)
chrX-38145105	c.3146_3147del	p.(Glu1049Glyfs*29)	Likely Pathogenic (PVS1, PM2)
chrX-38145105	c.3146_3149del	p.(Glu1049Glyfs*39)	Likely Pathogenic (PVS1, PM2)
chrX-38145040	c.3212dup	p.(Thr1072Aspfs*7)	Pathogenic (PVS1, PM2, PP1)
chrX-38145000	c.3248_3252del	p.(Glu1083Valfs*17)	Likely Pathogenic (PVS1, PM2)
chrX-38144991	c.3261del	p.(Val1088*)	Likely Pathogenic (PVS1, PM2)
chrX-38144976	c.3276del	p.(Gly1093Aspfs*3)	Likely Pathogenic (PVS1, PM2)
chrX-38144918	c.3334C>T	p.(Gln1112*)	Likely Pathogenic (PVS1, PM2)
chrX-38144857	c.3395dup	p.(Asn1132Lysfs*12)	Likely Pathogenic (PVS1, PM2)

The American College of Medical Genetics (ACMG) criteria for this study: PVS1: null variant (nonsense, frameshift, canonical ± 1 or 2 splice sites or initiation codon); PM2: frequency on gnomAD < 0.5% and no homozygous cases (if not: BS1); PP1: Cosegregation with disease verified; PP3: At least 1 predictive algorithm suggests pathogenicity (for splice variants, score ≤ −10%), if not: BP4; BP1: Missense variant in a gene for which primarily truncating variants are known to cause disease.

**Table 3:** The results of the data analysis on patients with cone/cone-dystrophy (CDs/CRDs) included in the study.

	*RPGR^ORF15^*
Male, No./Total No. (%)	36/36 (100)
Age of Onset	*n* = 18
1st decade	4
2nd decade	9
3rd decade	4
≥4th decade	1
High Myopia, No./Total No. (%)	14/33 (42.42)
Cataract and/or previous cataract surgery in at least one eye, No./Total No. (%)	4/33 (12.12)
^ǂ^ CD/CRD, No/No	15/21
Age at last visit, mean ± SD, y	*n* = 36; 43.97 ± 11.24
Light perception or no light perception in at least one eye, No./total No. (%)	1/35 (2.86)
^§^ BCVA at last visit, LogMar, Mean ± SD [Snellen Equivalent]	*n* = 35; 0.97 ± 0.71 [20/200]
Follow-up BCVA, No	*n* = 35 [range: 0–19 y]
0 y	11
1–5 y	14
6–10 y	9
>10 y	1
Estimation of BCVA decline	*n* = 24
Annual rate, LogMAR/year ± SD	0.04 ± 0.06
Mean regression slope ± SD	0.04 ± 0.08
Mean regression intercept ± SD	−0.90 ± 3.34
Binocular normal color vision, No./Total No. (%)	1/32 (3.12)
Bilateral undetectable ff-ERG, No./Total No. (%)	5/36 (13.89)
SW-FAF Phenotype type	*n* = 35
Group 1	6
Group 2	21
Group 3	8
Peripapillary sparing, No./Total No. (%)	10/35 (28.57)
Estimation of central hyperAF ring enlargement	*n* = 19
Horizontal diameter, µm/y	42.88 ± 48.98
Slope	43.16 ± 47.81
Intercept	228.60 ± 1689.24
Vertical diameter, µm/y	39.44 ± 40.90
Slope	39.36 ± 40.57
Intercept	−24.16 ± 1658.86
CRT, µm, mean ± SD	*n* = 35; 148.97 ± 27.17
Unilateral or bilateral ERM, No./Total No. (%)	2/35 (2.78)
Unilateral or bilateral iHRF, No./Total No. (%)	15/35 (42.86)

No.—Number; SD—Standard deviation; BCVA—Best corrected visual acuity; VF—Visual field; iHRF—Intraretinal hyper-reflective foci; ERM—Epiretinal membrane; ff-ERG—Full field electroretinogram; SW-FAF—Short-wavelength fundus autofluorescence; OCT—Optical coherence tomography; CRT—Central retinal thickness. ^ǂ^—Definition of CDs and CRDs was based on the ff-ERG; ^§^—For BCVA calculation, patients with or without light perception were excluded as this is not possible to convert to LogMar. Counting fingers and hand motions were converted to 2 and 3 LogMar, respectively [24].

**Table 4:** The stepwise linear regression analysis investigating the association between the best corrected visual acuity and several parameters included in the study.

	Univariate		Multivariate	
	β Coefficient	*p* Value	β Coefficient	*p* Value
Age	0.306	0.078	-	-
Decade of onset	−0.241	0.315	-	-
High Myopia	−0.094	0.609	-	-
ff-ERG	0.140	0.438	-	-
iHRF	−0.266	0.135	-	-
SW-FAF phenotype	0.353	0.044	0.092	0.573
Peripapillary sparing	−0.376	0.031	−0.385	0.019
CRT	−0.523	0.003	−0.456	0.006

ff-ERG—full-field electroretinogram, 0: residual responses, 1: undetectable; ERM—epiretinal membrane, 0: absent, 1: present; iHRF—intraretinal hyper-reflective foci, 0: absent, 1: present; SW-FAF phenotype:—1: the dimension of the central hypoautofluorescence is inferior to the 1 disc diameter (DD), 2: the dimension of the central hypoautofluorescence exceeds the 1 disc diameter (DD) but stays within the macula, 3: the central hypoautofluorescence extends beyond the macula; Peripapillary sparing—0: absent, 1: present; CRT—central retinal thickness.

## Data Availability

All relevant data are reported within the publication and its supplementary material.

## Supplementary Materials

The following supporting information can be downloaded at: https://www.mdpi.com/article/10.3390/ijms23137189/s1.

## References

[1] Hamel C.P. (2007). Cone Rod Dystrophies. Orphanet. J. Rare Dis..

[2] Meindl A., Dry K., Herrmann K., Manson F., Ciccodicola A., Edgar A., Carvalho M.R., Achatz H., Hellebrand H., Lennon A. (1996). A Gene (RPGR) with Homology to the RCC1 Guanine Nucleotide Exchange Factor Is Mutated in X-Linked Retinitis Pigmentosa (RP3). Nat. Genet..

[3] Sharon D., Sandberg M.A., Rabe V.W., Stillberger M., Dryja T.P., Berson E.L. (2003). RP2 and RPGR Mutations and Clinical Correlations in Patients with X-Linked Retinitis Pigmentosa. Am. J. Hum. Genet..

[4] Talib M., van Schooneveld M.J., Thiadens A.A., Fiocco M., Wijnholds J., Florijn R.J., Schalij-Delfos N.E., van Genderen M.M., Putter H., Cremers F.P.M. (2019). Clinical and Genetic Characteristics of Male Patients with Rpgr-Associated Retinal Dystrophies: A Long-Term Follow-up Study. Retina.

[5] Megaw R.D., Soares D.C., Wright A.F. (2015). RPGR: Its Role in Photoreceptor Physiology, Human Disease, and Future Therapies. Exp. Eye Res..

[6] Schmid F., Glaus E., Cremers F.P.M., Kloeckener-Gruissem B., Berger W., Neidhardt J. (2010). Mutation- and Tissue-Specific Alterations of RPGR Transcripts. Investig. Ophthalmol. Vis. Sci..

[7] Vössing C., Atigbire P., Eilers J., Markus F., Stieger K., Song F., Neidhardt J. (2021). The Major Ciliary Isoforms of RPGR Build Different Interaction Complexes with INPP5E and RPGRIP1L. Int. J. Mol. Sci..

[8] Vervoort R., Lennon A., Bird A.C., Tulloch B., Axton R., Miano M.G., Meindl A., Meitinger T., Ciccodicola A., Wright A.F. (2000). Mutational Hot Spot within a New RPGR Exon in X-Linked Retinitis Pigmentosa. Nat. Genet..

[9] Remans K., Bürger M., Vetter I.R., Wittinghofer A. (2014). C2 Domains as Protein-Protein Interaction Modules in the Ciliary Transition Zone. Cell Rep..

[10] Hong D.H., Pawlyk B.S., Shang J., Sandberg M.A., Berson E.L., Li T. (2000). A Retinitis Pigmentosa GTPase Regulator (RPGR)-Deficient Mouse Model for X-Linked Retinitis Pigmentosa (RP3). Proc. Natl. Acad. Sci. USA.

[11] Hong D.-H., Pawlyk B., Sokolov M., Strissel K.J., Yang J., Tulloch B., Wright A.F., Arshavsky V.Y., Li T. (2003). RPGR Isoforms in Photoreceptor Connecting Cilia and the Transitional Zone of Motile Cilia. Investig. Ophthalmol. Vis. Sci..

[12] Mavlyutov T.A., Zhao H., Ferreira P.A. (2002). Species-Specific Subcellular Localization of RPGR and RPGRIP Isoforms: Implications for the Phenotypic Variability of Congenital Retinopathies among Species. Hum. Mol. Genet..

[13] Ruddle J.B., Ebenezer N.D., Kearns L.S., Mulhall L.E., Mackey D.A., Hardcastle A.J. (2009). RPGR ORF15 Genotype and Clinical Variability of Retinal Degeneration in an Australian Population. Br. J. Ophthalmol..

[14] De Silva S.R., Arno G., Robson A.G., Fakin A., Pontikos N., Mohamed M.D., Bird A.C., Moore A.T., Michaelides M., Webster A.R. (2021). The X-Linked Retinopathies: Physiological Insights, Pathogenic Mechanisms, Phenotypic Features and Novel Therapies. Prog. Retin. Eye Res..

[15] Thiadens A.A.H.J., Soerjoesing G.G., Florijn R.J., Tjiam A.G., den Hollander A.I., van den Born L.I., Riemslag F.C., Bergen A.A.B., Klaver C.C.W. (2011). Clinical Course of Cone Dystrophy Caused by Mutations in the RPGR Gene. Graefes Arch. Clin. Exp. Ophthalmol..

[16] Zahid S., Khan N., Branham K., Othman M., Karoukis A.J., Sharma N., Moncrief A., Mahmood M.N., Sieving P.A., Swaroop A. (2013). Phenotypic Conservation in Patients with X-Linked Retinitis Pigmentosa Caused by RPGR Mutations. JAMA Ophthalmol..

[17] Richards S., Aziz N., Bale S., Bick D., Das S., Gastier-Foster J., Grody W.W., Hegde M., Lyon E., Spector E. (2015). Standards and Guidelines for the Interpretation of Sequence Variants: A Joint Consensus Recommendation of the American College of Medical Genetics and Genomics and the Association for Molecular Pathology. Genet. Med..

[18] García-Hoyos M., Garcia-Sandoval B., Cantalapiedra D., Riveiro R., Lorda-Sánchez I., Trujillo-Tiebas M.J., Rodriguez de Alba M., Millan J.M., Baiget M., Ramos C. (2006). Mutational Screening of the RP2 and RPGR Genes in Spanish Families with X-Linked Retinitis Pigmentosa. Investig. Ophthalmol. Vis. Sci..

[19] Mawatari G., Fujinami K., Liu X., Yang L., Yokokawa Y.-F., Komori S., Ueno S., Terasaki H., Katagiri S., Hayashi T. (2019). Clinical and Genetic Characteristics of 14 Patients from 13 Japanese Families with RPGR-Associated Retinal Disorder: Report of Eight Novel Variants. Hum. Genome. Var..

[20] Demirci F.Y.K., Gupta N., Radak A.L., Rigatti B.W., Mah T.S., Milam A.H., Gorin M.B. (2005). Histopathologic Study of X-Linked Cone-Rod Dystrophy (CORDX1) Caused by a Mutation in the RPGR Exon ORF15. Am. J. Ophthalmol..

[21] Olm M.A.K., Marson F.A.L., Athanazio R.A., Nakagawa N.K., Macchione M., Loges N.T., Omran H., Rached S.Z., Bertuzzo C.S., Stelmach R. (2019). Severe Pulmonary Disease in an Adult Primary Ciliary Dyskinesia Population in Brazil. Sci. Rep..

[22] Shu X., Black G.C., Rice J.M., Hart-Holden N., Jones A., O’Grady A., Ramsden S., Wright A.F. (2007). RPGR Mutation Analysis and Disease: An Update. Hum. Mutat..

[23] Bader I., Brandau O., Achatz H., Apfelstedt-Sylla E., Hergersberg M., Lorenz B., Wissinger B., Wittwer B., Rudolph G., Meindl A. (2003). X-Linked Retinitis Pigmentosa: RPGR Mutations in Most Families with Definite X Linkage and Clustering of Mutations in a Short Sequence Stretch of Exon ORF15. Investig. Ophthalmol. Vis. Sci..

[24] Holladay J.T. (1997). Proper Method for Calculating Average Visual Acuity. J. Refract. Surg..

[25] Birtel J., Eisenberger T., Gliem M., Müller P.L., Herrmann P., Betz C., Zahnleiter D., Neuhaus C., Lenzner S., Holz F.G. (2018). Clinical and Genetic Characteristics of 251 Consecutive Patients with Macular and Cone/Cone-Rod Dystrophy. Sci. Rep..

[26] Gill J.S., Georgiou M., Kalitzeos A., Moore A.T., Michaelides M. (2019). Progressive Cone and Cone-Rod Dystrophies: Clinical Features, Molecular Genetics and Prospects for Therapy. Br. J. Ophthalmol..

[27] Stenson P.D., Ball E.V., Mort M., Phillips A.D., Shiel J.A., Thomas N.S.T., Abeysinghe S., Krawczak M., Cooper D.N. (2003). Human Gene Mutation Database (HGMD): 2003 Update. Hum. Mutat..

[28] Sharon D., Bruns G.A., McGee T.L., Sandberg M.A., Berson E.L., Dryja T.P. (2000). X-Linked Retinitis Pigmentosa: Mutation Spectrum of the RPGR and RP2 Genes and Correlation with Visual Function. Investig. Ophthalmol. Vis. Sci..

[29] Ayyagari R., Demirci F.Y., Liu J., Bingham E.L., Stringham H., Kakuk L.E., Boehnke M., Gorin M.B., Richards J.E., Sieving P.A. (2002). X-Linked Recessive Atrophic Macular Degeneration from RPGR Mutation. Genomics.

[30] Ebenezer N.D., Michaelides M., Jenkins S.A., Audo I., Webster A.R., Cheetham M.E., Stockman A., Maher E.R., Ainsworth J.R., Yates J.R. (2005). Identification of Novel RPGR ORF15 Mutations in X-Linked Progressive Cone-Rod Dystrophy (XLCORD) Families. Investig. Ophthalmol. Vis. Sci..

[31] Chiang J.P.-W., Lamey T., McLaren T., Thompson J.A., Montgomery H., De Roach J. (2015). Progress and Prospects of Next-Generation Sequencing Testing for Inherited Retinal Dystrophy. Expert. Rev. Mol. Diagn..

[32] Chiang J.P.W., Lamey T.M., Wang N.K., Duan J., Zhou W., McLaren T.L., Thompson J.A., Ruddle J., De Roach J.N. (2018). Development of High-Throughput Clinical Testing of RPGR ORF15 Using a Large Inherited Retinal Dystrophy Cohort. Investig. Ophthalmol. Vis. Sci..

[33] Linari M., Ueffing M., Manson F., Wright A., Meitinger T., Becker J. (1999). The Retinitis Pigmentosa GTPase Regulator, RPGR, Interacts with the Delta Subunit of Rod Cyclic GMP Phosphodiesterase. Proc. Natl. Acad. Sci. USA.

[34] Anand M., Khanna H. (2012). Ciliary Transition Zone (TZ) Proteins RPGR and CEP290: Role in Photoreceptor Cilia and Degenerative Diseases. Expert. Opin. Ther. Targets.

[35] Sun X., Park J.H., Gumerson J., Wu Z., Swaroop A., Qian H., Roll-Mecak A., Li T. (2016). Loss of RPGR Glutamylation Underlies the Pathogenic Mechanism of Retinal Dystrophy Caused by TTLL5 Mutations. Proc. Natl. Acad. Sci. USA.

[36] Hong D.-H., Pawlyk B.S., Adamian M., Li T. (2004). Dominant, Gain-of-Function Mutant Produced by Truncation of RPGR. Investig. Ophthalmol. Vis. Sci..

[37] Fahim A.T., Bowne S.J., Sullivan L.S., Webb K.D., Williams J.T., Wheaton D.K., Birch D.G., Daiger S.P. (2011). Allelic Heterogeneity and Genetic Modifier Loci Contribute to Clinical Variation in Males with X-Linked Retinitis Pigmentosa Due to RPGR Mutations. PLoS ONE.

[38] Körschen H.G., Illing M., Seifert R., Sesti F., Williams A., Gotzes S., Colville C., Müller F., Dosé A., Godde M. (1995). A 240 KDa Protein Represents the Complete Beta Subunit of the Cyclic Nucleotide-Gated Channel from Rod Photoreceptor. Neuron.

[39] Pearring J.N., Martínez-Márquez J., Willer J.R., Lieu E.C., Salinas R.Y., Arshavsky V.Y. (2021). The GARP Domain of the Rod CNG Channel’s B1-Subunit Contains Distinct Sites for Outer Segment Targeting and Connecting to the Photoreceptor Disk Rim. J. Neurosci..

[40] Nassisi M., Smirnov V.M., Solis Hernandez C., Mohand-Saïd S., Condroyer C., Antonio A., Kühlewein L., Kempf M., Kohl S., Wissinger B. (2021). CNGB1-Related Rod-Cone Dystrophy: A Mutation Review and Update. Hum. Mutat..

[41] Rinaldi C., Donato L., Alibrandi S., Scimone C., D’Angelo R., Sidoti A. (2021). Oxidative Stress and the Neurovascular Unit. Life.

[42] Scimone C., Donato L., Alibrandi S., Vadalà M., Giglia G., Sidoti A., D’Angelo R. (2021). N-Retinylidene-N-Retinylethanolamine Adduct induces Expression of Chronic Inflammation Cytokines in Retinal Pigment Epithelium Cells. Exp. Eye Res..

[43] Donato L., Abdalla E.M., Scimone C., Alibrandi S., Rinaldi C., Nabil K.M., D’Angelo R., Sidoti A. (2021). Impairments of Photoreceptor Outer Segments Renewal and Phototransduction Due to a Peripherin Rare Haplotype Variant: Insights from Molecular Modeling. Int. J. Mol. Sci..

[44] Khateb S., Mohand-Saïd S., Nassisi M., Bonnet C., Roux A.-F., Andrieu C., Antonio A., Condroyer C., Zeitz C., Devisme C. (2020). Phenotypic Characteristics of Rod-Cone Dystrophy Associated with Myo7a Mutations in A Large French Cohort. Retina.

[45] Khateb S., Nassisi M., Bujakowska K.M., Méjécase C., Condroyer C., Antonio A., Foussard M., Démontant V., Mohand-Saïd S., Sahel J.-A. (2019). Longitudinal Clinical Follow-up and Genetic Spectrum of Patients With Rod-Cone Dystrophy Associated With Mutations in PDE6A and PDE6B. JAMA Ophthalmol..

[46] Greenstein V.C., Duncker T., Holopigian K., Carr R.E., Greenberg J.P., Tsang S.H., Hood D.C. (2012). Structural and Functional Changes Associated with Normal and Abnormal Fundus Autofluorescence in Patients with Retinitis Pigmentosa. Retina.

[47] Packer O., Hendrickson A.E., Curcio C.A. (1989). Photoreceptor Topography of the Retina in the Adult Pigtail Macaque (Macaca Nemestrina). J. Comp. Neurol..

[48] Vrabec F. (1966). The Temporal Raphe of the Human Retina. Am. J. Ophthalmol..

[49] Birtel J., Gliem M., Herrmann P., MacLaren R.E., Bolz H.J., Charbel Issa P. (2020). Peripapillary Sparing in Autosomal Recessive Bestrophinopathy. Ophthalmol. Retina.

[50] Burke T.R., Rhee D.W., Smith R.T., Tsang S.H., Allikmets R., Chang S., Lazow M.A., Hood D.C., Greenstein V.C. (2011). Quantification of Peripapillary Sparing and Macular Involvement in Stargardt Disease (STGD1). Investig. Ophthalmol. Vis. Sci..

[51] Garg A., Lee W., Sengillo J.D., Allikmets R., Garg K., Tsang S.H. (2017). Peripapillary Sparing in RDH12-Associated Leber Congenital Amaurosis. Ophthalmic. Genet..

[52] Nassisi M., Mohand-Saïd S., Andrieu C., Antonio A., Condroyer C., Méjécase C., Dhaenens C.-M., Sahel J.-A., Zeitz C., Audo I. (2019). Peripapillary Sparing with Near Infrared Autofluorescence Correlates with Electroretinographic Findings in Patients With Stargardt Disease. Investig. Ophthalmol. Vis. Sci..

[53] Cideciyan A.V., Swider M., Aleman T.S., Sumaroka A., Schwartz S.B., Roman M.I., Milam A.H., Bennett J., Stone E.M., Jacobson S.G. (2005). ABCA4-Associated Retinal Degenerations Spare Structure and Function of the Human Parapapillary Retina. Investig. Ophthalmol. Vis. Sci..

[54] Neidhardt J., Glaus E., Lorenz B., Netzer C., Li Y., Schambeck M., Wittmer M., Feil S., Kirschner-Schwabe R., Rosenberg T. (2008). Identification of Novel Mutations in X-Linked Retinitis Pigmentosa Families and Implications for Diagnostic Testing. Mol. Vis..

[55] Fokkema I.F.A.C., Taschner P.E.M., Schaafsma G.C.P., Celli J., Laros J.F.J., den Dunnen J.T. (2011). LOVD v.2.0: The next Generation in Gene Variant Databases. Hum. Mutat..

[56] Adzhubei I.A., Schmidt S., Peshkin L., Ramensky V.E., Gerasimova A., Bork P., Kondrashov A.S., Sunyaev S.R. (2010). A Method and Server for Predicting Damaging Missense Mutations. Nat. Methods.

[57] Sim N.-L., Kumar P., Hu J., Henikoff S., Schneider G., Ng P.C. (2012). SIFT Web Server: Predicting Effects of Amino Acid Substitutions on Proteins. Nucleic Acids Res..

[58] Schwarz J.M., Cooper D.N., Schuelke M., Seelow D. (2014). MutationTaster2: Mutation Prediction for the Deep-Sequencing Age. Nat. Methods.

[59] Casper J., Zweig A.S., Villarreal C., Tyner C., Speir M.L., Rosenbloom K.R., Raney B.J., Lee C.M., Lee B.T., Karolchik D. (2018). The UCSC Genome Browser Database: 2018 Update. Nucleic Acids Res..

[60] Kent W.J. (2002). BLAT—The BLAST-like Alignment Tool. Genome Res..

[61] McCulloch D.L., Marmor M.F., Brigell M.G., Hamilton R., Holder G.E., Tzekov R., Bach M. (2015). ISCEV Standard for Full-Field Clinical Electroretinography (2015 Update). Doc. Ophthalmol..

