# Supplementary material for: Retrospective Natural History Study of RPGR-Related Cone- and Cone-Rod Dystrophies While Expanding the Mutation Spectrum of the Disease
Source: Int J Mol Sci. 2022 Jun 28;23(13):7189. doi: 10.3390/ijms23137189 (PMC9266815; doi:10.3390/ijms23137189)
Supplement: Supplementary file 1 [file ijms-23-07189-s001.zip › ijms-1657011-supplementary.pdf]

RPGR-related Cone- and Cone-Rod Dystrophies: retrospective natural history study and genotype-phenotype correlation.

Marco Nassisi, Giuseppe De Bartolo, Saddek Mohand-Said, Christel Condroyer, Aline Antonio, Marie-Elise Lancelot, Kinga Bujakowska, Vasily Smirnov, Thomas Pugliese, John Neidhardt, José-Alain Sahel, Christina Zeitz*, Isabelle Audo*.

* These authors contributed equally

Supplementary material

Figure S1. Pedigree of the families for whom segregation analysis was available.

Figure S2. Patients excluded from quantitative SW-FAF analysis

Table S1. *In silico* analysis *RPGR*^{ORF15} novel missense variant.

Table S2. *RPGR*^{ORF15} conservation analysis for the novel missense variant.

Table S3. Clinical data of the cohort.

Table S4. List of primers used for *RPGR*^{ORF15} exon 15 Sanger sequencing.

Table S5. Agreement between eyes.

Figure S1. Pedigree of the families for whom segregation analysis was available. The black arrowheads indicate the proband.

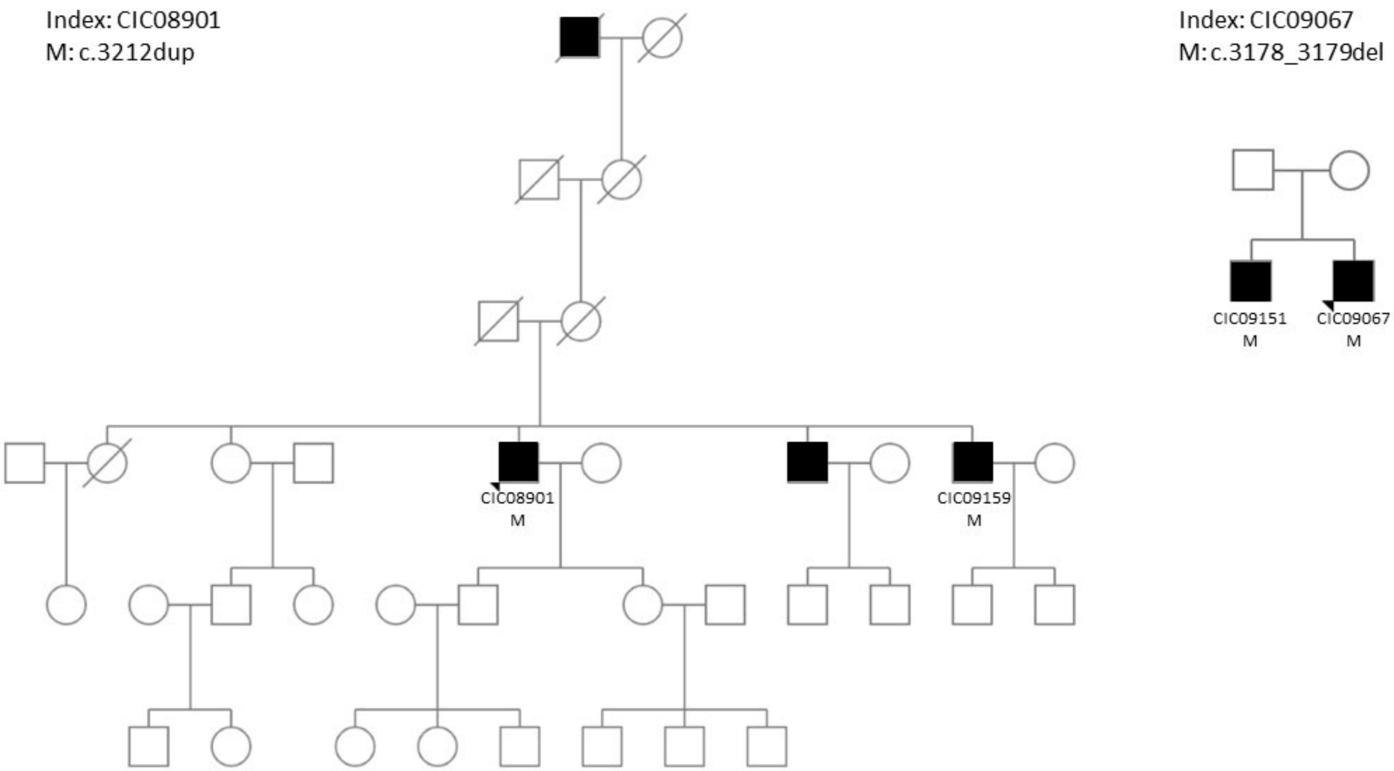

Figure S2. Patients excluded from quantitative SW-FAF analysis as the hyperautofluorescent ring encircling the dystrophic retina either included the optic nerve head or disappeared during the follow-up.

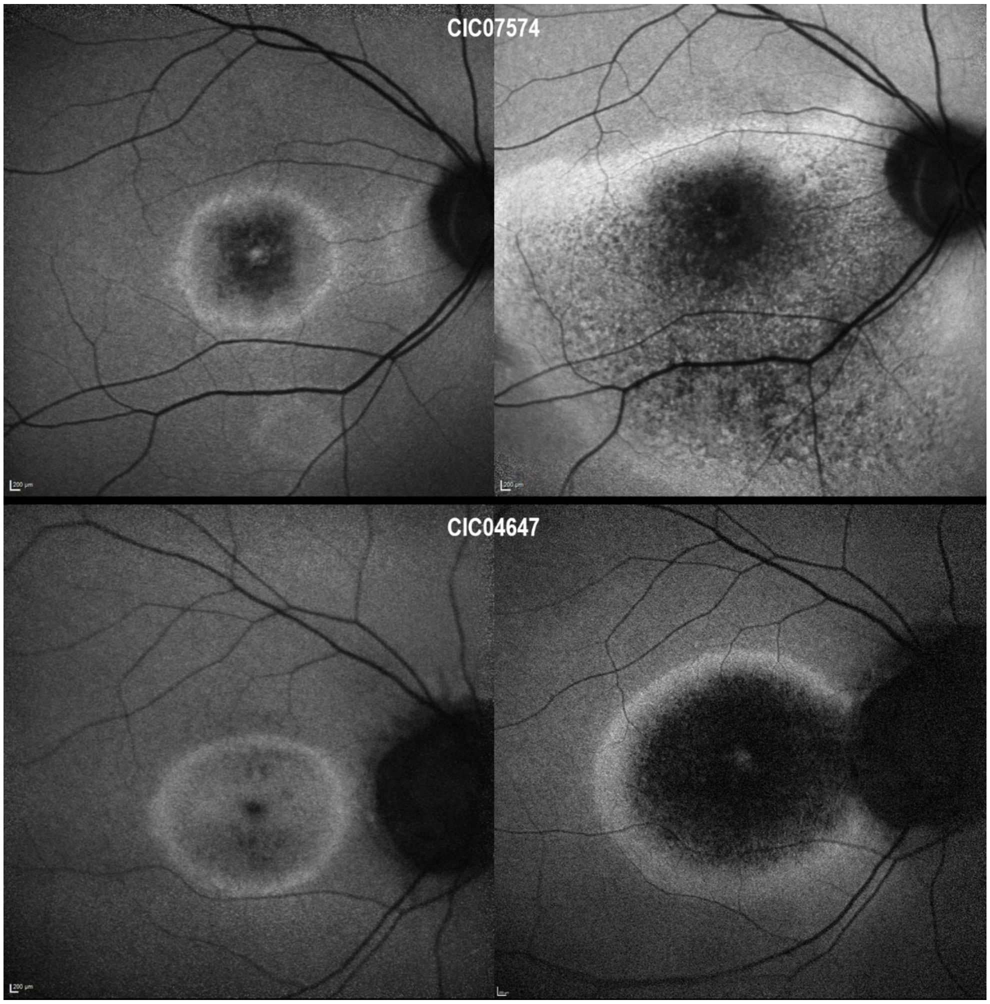

Table S1. *In silico* analysis performed on the $RPGR^{ORF15}$ missense variant included in the study. PhyloP range: -14.1;6.4. Grantham score range: 0;215.

Genomic start position (hg19)	cDNA (NM_001034853.1)	Protein change	rs#; gnomAD: frequency (allele count/total alleles/number of homozygous)	PhyloP	Grantham	Pathogenic prediction for missense changes			Effect on splicing prediction		
						SIFT (score)	MutuationTaster (prob)	PolyPhen 2 (score)	MaxEnt	NNSplice	SSF
38145574	c.2678G>T	p.(Gly893 Val)	no rs#; gnomAD: absent	-0,28	109	Tolerated (0.24)	disease causing (0.682)	possibly damaging (0.743)	-	-	-

Table S2. Conservation analysis of the novel missense variant c.2678G>T p.(Gly893Val) on *RPGR*^{ORF15}, described in the study. This analysis was performed using the University of California Santa Cruz (UCSC) Genome Browser, available at https://genome.ucsc.edu/index.html. The interested residue is highlighted in red.

Nucleotide Genomic position (hg38 – NM_001034853.1)	**38145574**	Nucleotide genomic position (hg19)	**48835341**	Nucleotide genomic position (hg19)	**48835341**
AA position	**893**	AA position	**893**	AA position	**893**
Physiological residue	**G**	Physiological residue	**G**	Physiological residue	**G**
Altered residue	**V**	Altered residue	**V**	Altered residue	**V**
Human	EEEGEGEGEEE	Domestic goat	-----------	Budgerigar	EEE-EGV--KK
Chimp	-----------	Horse	-----------	Parrot	EKG-DCK--GK
Gorilla	-----------	White rhinoceros	NGE-GEGER--	Scarlet macaw	GKG-DCK--GK
Orangutan	-----------	Cat	GEE-EGKGEVE	Rock pigeon	CEG-VPKEYAK
Gibbon	-----------	Dog	GEE-EGKGEVE	Mallard duck	GEP--EMEDLQ
Rhesus	KE--GRRREEE	Ferret	-----------	Chicken	QEQ-RGKQGAK
Crab-eating Macaque	-----------	Panda	EGE-GEREGEK	Turkey	RRQ-EEEEEAE
Baboon	-----------	Pacific walrus	GEE-EGEGEAE	American alligator	D---EGEDDGE
Green Monkey	-----------	Weddell seal	EEG-GEGEVEE	Green seaturtle	REE-KGSMVGD
Marmoset	-----------	Black flying-fox	--D-DGEEEEE	Painted turtle	EAE-EGGEEDE
Squirrel Monkey	EEEGEREEEGE	Megabat	-----------	Chinese softshell turtle	EED-KSEEEEG
Bushbaby	EG---------	David's myotis (bat)	-----------	Spiny softshell turtle	EED-KSEEEED
Chinese tree shrew	EEEEEEEEGEG	Big brown bat	E---GGEEGEE	Lizard	EKE-EKENEEG
Squirrel Monkey	KEEKEERGKGE	Hedgehog	GGE-EEGGEEE	X_tropicalis	EEE--EDEEEE
Egyptian jerboa	EEEEEKT--EE	Shrew	EEG-EEGADED	Coelacanth	EEK-DAESADE
Prairie	EEE------EE	Star-nosed mole	EEG-EGDQGEE	Tetraodon	-----------
Chinese hamster	EGE------EE	Elephant	-----------	Fugu	-----------
Golden hamster	EGE------EG	Cape elephant shrew	GEG-EGEGEEE	Yellowbelly pufferfish	-----------
Mouse	EGE------EE	Manatee	GEE-QKEREEE	Nile tilapia	SKR-DTESDLE
Rat	DEE------ED	Cape golden mole	-----------	Princess of Burundi	SKR-DTESDLD
Naked mole-rat	KEG--------	Tenrec	EE---GEGNDE	Burton's mouthbreeder	SKR-DTESDLE
Guinea pig	-----------	Aardvark	-----------	Zebra mbuna	SKR-DTESDLE
Chinchilla	KEGEEKE---G	Armadillo	EEE-EAEREEE	Pundamilia nyererei	SKR-DTESDLE
Brush-tailed rat	EEGEEEE--EE	Opossum	RKK--ERSRNG	Medaka	-----------
Rabbit	-----------	Tasmanian devil	SKK--ERSRNG	Southern platyfish	N-----RGRRE
Pika	EEE------EG	Wallaby	-----------	Stickleback	SEE-EVDSGEE
Pig	DGE-EGEGGEE	Platypus	EEE-GEEEEEL	Atlantic cod	-----------
Alpaca	----EGQEEEG	Saker falcon	KKR-EKE-HEK	Zebrafish	R---EKESVAE
Bactrian camel	EEE-EGEEEEG	Peregrine falcon	-----------	Mexican tetra (cavefish)	EES-VKE-DHE
Dolphin	EEE-EEGGEEE	Collared flycatcher	-AG-E-NECEQ	Spotted gar	SRT-EVE----
Killer whale	EEE-EEGGEEE	White-throated sparrow	EMR-EGGEEER	Lamprey	DRG---GEDDA
Tibetan antelope	-----------	Medium ground finch	GMG-KWG--EG		
Cow	-----------	Zebra finch	GMR-ETEEEQR		
Sheep	GGG-EEGGGEE	Tibetan ground jay	-----------		

Table S3. Clinical data from cone and cone-rod (CD and CRD) patients carrying variants on *RPGR*^{ORF15}.

ID	Gender	Age of last visit	Decade of onset	Color Vision (Altered Axis)	Comorbidities	BCVA OD	BCVA OS	ff-ERG	SW-FAF	SW-FAF pattern	OCT	ERM	iHRF
CIC03862	M	9	1st decade	Normal	None	0	0	Normal Scotopic; photopic reduced	Normal	1A	Central dystrophic changes in the EZ	no	no
CIC01418	M	25	2nd decade	Dyschromatopsia without definite axis	None	1.2	1.3	Both reduced (photopic +)	Patchy hypoauofluorescence along the temporal vascular arcades. Homogeneous hypoautofluorescence of the posterior pole surrounded by halo of hyperautofluorescence	3B	Generalized thinning of the retina	no	no
782819	M	37		Deutan OD / Protan OS	High Myopia	0.7	0.7	Scotopic reduced; photopic undetectable	Peripapillary atrophy; Grainy central hypoautofluorescence surrounded by hyperfluorescent ring	2B	Posterior Staphyloma; Central RPE/outer retina atrophy; Preservation of all retinal layers around the lesion	no	no
CIC09352	M	51		Not performed, vision too low	Glaucoma	1.3	1.2	Scotopic reduced; photopic undetectable	Central circular area of deep hypoautofluorescence, surrounded by halo of hyperautofluorescence	2A	Central RPE/outer retina atrophy; Preservation of all retinal layers around the lesion	no	no
CIC04404	M	36	2nd decade	Not performed, vision too low	High Myopia; Previous cataract surgery OO	2	1.6	Normal Scotopic; photopic reduced	Peripapillary atrophy and focal areas of hypoautofluorescence nasally; central circular area of deep hypoautofluorescence, surrounded by halo of hyperautofluorescence	2B	Posterior Staphyloma; Central RPE/outer retina atrophy; Preservation of all retinal layers around the lesion	no	no
CIC02893	M	53	4th decade	Deutan	None	0.5	0.5	Both reduced (photopic +)	Peripapillary atrophy; Central area of	2B	Central preservation of the outer retinal layers,	no	no

			(color vision)						isoautofluorescence surrounded by ring of granular hypoautofluorescence encircled by an hyperautofluorescent ring		surrounded by areas of dystrophic alterations of outer retinal layers with HRDs; preservation of the retinal layers in the periphery of the acquisition.		
CIC02863	M	35	2nd decade	Dyschromatopsia without definite axis	None	1	0.8	Undetectable	Diffuse hypoautofluorescence	2B	Generalized RPE/Outer retina atrophy	no	yes
CIC07494	M	61	1st decade	Not performed, vision too low	Previous cataract surgery OO	3	3	Undetectable	Diffuse hypoautofluorescence	3B	Generalized RPE/Outer retina atrophy	no	yes
CIC06631	M	34	2nd decade	Not performed, vision too low	High Myopia			Scotopic reduced; photopic undetectable	Peripapillary atrophy; central circular area of deep hypoautofluorescence, surrounded by halo of hyperautofluorescence	2B	Central RPE/outer retina atrophy; Preservation of all retinal layers around the lesion	no	no
CIC03560	M	55	2nd decade	Tritan	None	0.4	0.2	Normal Scotopic; photopic reduced	Central area of isoautofluorescence surrounded by ring of granular hypoautofluorescence encircled by an hyperautofluorescent ring	2A	Central area of isoautofluorescence surrounded by ring of granular hypoautofluorescence encircled by an hyperautofluorescent ring	no	no
CIC06538	M	51	1st decade	Deutan OD/ Deutan and Tritan OS	High Myopia	1.1	1.2	Scotopic reduced; photopic undetectable	Central area of isoautofluorescence surrounded by ring of granular hypoautofluorescence encircled by an hyperautofluorescent ring	2B	Central preservation of the outer retinal layers, surrounded by areas of dystrophic alterations of outer retinal layers; preservation of the retinal layers in the periphery of the acquisition.	no	yes

CIC04447	M	38	2nd decade	Dyschromatopsia without definite axis	High Myopia	1	1.1	Scotopic reduced; photopic undetectable	Peripapillary atrophy; Lacquer cracks; grainy central hypoautofluorescence surrounded by hyperfluorescent ring	3B	Posterior Staphyloma; Central RPE/outer retina atrophy; Preservation of all retinal layers around the lesion	no	no
CIC04647	M	44		Dyschromatopsia without definite axis	High Myopia	0.7	0.6	Scotopic reduced; photopic undetectable	Peripapillary atrophy; Grainy central hypoautofluorescence surrounded by hyperfluorescent ring	2B	Posterior Staphyloma; Central RPE/outer retina atrophy; Preservation of all retinal layers around the lesion	no	no
CIC09451	M	48		Dyschromatopsia without definite axis	High Myopia	1.3	1.3	Scotopic reduced; photopic undetectable	Peripapillary atrophy; Grainy central hypoautofluorescence surrounded by hyperfluorescent ring	1B	Central RPE/outer retina atrophy; Preservation of all retinal layers around the lesion	no	no
CIC07574	M	39	2nd decade	Not performed, vision too low	None	1	1	Scotopic reduced; photopic undetectable	Central area of hypoautofluorescence with granular hypo/hyperautofluorescence extending towards the inferior, temporal and nasal directions	3B	Central outer retina atrophy	no	yes
CIC07658	M	46		Tritan	High Myopia	0.3	0.3	Undetectable	Semicircular area of patchy hypoautofluorescence extending from around the optic disc towards the inferior temporal arcade and involving the central macula (with foveal sparing); This area is surrounded by an hyperautofluorescent halo.	3B	Generalized retinal thinning with foveal sparing	no	yes
CIC07798	M	39		Deutan and Protan	None	1	0.9	Normal Scotopic; photopic reduced	Central circular area of deep hypoautofluorescence,	1B	Central RPE/outer retina atrophy; Preservation of	no	no

									surrounded by halo of hyperautofluorescence		all retinal layers around the lesion		
CIC08152	M	35		Not performed, vision too low	None	1	1.2	Normal Scotopic; photopic reduced	Peripapillary atrophy; central circular area of deep hypoautofluorescence, surrounded by halo of hyperautofluorescence	2B	Central RPE/outer retina atrophy; Preservation of all retinal layers around the lesion	no	no
CIC8901	M	73		Not performed, vision too low	None	1.2	1.1	Scotopic reduced; photopic undetectable	Central circular area of deep hypoautofluorescence, surrounded by halo of hyperautofluorescence	2B	Central RPE/outer retina atrophy; Preservation of all retinal layers around the lesion	no	no
CIC8918	M	47		Deutan	None	1.3	1.3	Normal Scotopic; photopic reduced	Central area of hypoautofluorescence surrounded by halo of hyperautofluorescence	1A	Central EZ interruption	no	no
CIC9067	M	48			High Myopia	0.2	0.4	Normal Scotopic; photopic reduced	Peripapillary atrophy; central area of hypoautofluorescence, surrounded by halo of hyperautofluorescence	2B	Dystrophic/atrophic changes of the EZ in the center	no	yes
CIC8950	M	47		Dyschromatopsia without definite axis	pseudophakic OU, retinal detachment OS	0.9	1.2	Normal Scotopic; photopic reduced	RE: Peripapillary atrophy; central area of hypoautofluorescence, surrounded by halo of hyperautofluorescence. LE: Large area of chorioretinal atrophy extending from the macular towards the inferior periphery.	2B	RE: Central RPE/outer retina atrophy; Preservation of all retinal layers around the lesion; HRDs. LE: Rearrangements of the retinal structure with loss of physiologic retinal profile	no	yes
CIC09151	M	48				0.4	0.5	Scotopic reduced; photopic undetectable	Central area of isoautofluorescence surrounded by ring of granular hypoautofluorescence	2A	Central preservation of the outer retinal layers, surrounded by areas of dystrophic alterations of outer retinal layers with HRDs; preservation of the	no	no

									encircled by an hyperautofluorescent ring		retinal layers in the periphery of the acquisition.		
CIC01063	M	56		Not performed, vision too low	High myopia, glaucoma	LP	LP	Undetectable	Diffuse hypoautofluorescence	3B	Posterior staphyloma; diffuse atrophy of the outer retinal layers	no	no
CIC10466	M	51			None	0.3	0.3	Normal Scotopic; photopic reduced	Central circular area of hypoautofluorescence, surrounded by halo of hyperautofluorescence	2A	Central RPE/outer retina atrophy; Preservation of all retinal layers around the lesion	no	no
CIC09949	M	55	2nd decade	Not performed, vision too low		3	3	Undetectable	peripapillary and inferotemporal patchy hypoautofluorescence; diffuse granular hypoautofluorescence	3B	Central RPE/outer retina atrophy; Preservation of all retinal layers around the lesion	yes	yes
CIC10733	M	46		Tritan	None	0.3	0.5	Normal Scotopic; photopic reduced	Central area of isoautofluorescence surrounded by ring of granular hypoautofluorescence encircled by an hyperautofluorescent ring	2A	Central preservation of the outer retinal layers, surrounded by areas of dystrophic alterations of outer retinal layers with HRDs; preservation of the retinal layers in the periphery of the acquisition.	no	yes
CIC08066	M	28	1st decade	Dyschromatopsia without definite axis	High Myopia	0.2	0.2	Normal Scotopic; photopic reduced	Central area of hypoautofluorescence surrounded by halo of hyperautofluorescence	1A	Central EZ interruption	no	no
CIC04835	M	40	3rd decade	Protan	High Myopia	0.4	0.7	Normal Scotopic; photopic undetectable	Central area of isoautofluorescence surrounded by ring of granular hypoautofluorescence	2B	Central preservation of the outer retinal layers, surrounded by areas of dystrophic alterations of outer retinal layers; preservation of the retinal	no	yes

									encircled by an hyperautofluorescent ring		layers in the periphery of the acquisition.		
CIC03515	M	47	3rd decade	Dyschromatopsia without definite axis		0.2	0.3	Normal Scotopic; photopic reduced	Central area of hypoautofluorescence surrounded by halo of hyperautofluorescence	1B	Central EZ interruption	no	yes
CIC09159	M	57			Previous cataract surgery in OO	2	2	Scotopic reduced; photopic undetectable	Central area of isoautofluorescence surrounded by ring of granular hypoautofluorescence encircled by an hyperautofluorescent ring	2B	Central preservation of the outer retinal layers, surrounded by areas of dystrophic alterations of outer retinal layers with HRDs; preservation of the retinal layers in the periphery of the acquisition.	no	yes
CIC07400	M	38	3rd decade	Protan	None	0.7	0.6	Normal Scotopic; photopic reduced	Central area of isoautofluorescence surrounded by ring of granular hypoautofluorescence encircled by an hyperautofluorescent ring	2A	Central preservation of the outer retinal layers, surrounded by areas of dystrophic alterations of outer retinal layers with HRDs; preservation of the retinal layers in the periphery of the acquisition.	no	yes
CIC09653	M	39		Not performed, vision too low	High Myopia	1.5	1.3	Scotopic reduced; photopic undetectable					
CIC04850	M	39	3rd decade	Dyschromatopsia without definite axis (OD)/ normal (OS)	None	1.1	0.3	Scotopic highly reduced; photopic undetectable	Semicircular area of patchy hypoautofluorescence extending from around the optic disc towards the inferior temporal arcade; Central circular area of hypoautofluorescence	3B	Central RPE/outer retina atrophy; Preservation of all retinal layers around the lesion	no	yes

									surrounded by halo of hyperautofluorescence.				
CIC01503	M	41		Protan	High Myopia	0.9	0.7	Normal Scotopic; photopic undetectable	Peripapillary atrophy; Grainy central hypoautofluorescence surrounded by hyperfluorescent ring	2B	Posterior Staphyloma; Central RPE/outer retina atrophy; Preservation of all retinal layers around the lesion	no	yes
1659193	M	47	2nd decade	Protan	None	1	1	Scotopic reduced; photopic undetectable	Central area of hypoautofluorescence surrounded by halo of hyperautofluorescence	2A	Central RPE/outer retina atrophy; Preservation of all retinal layers around the lesion	yes	yes

OCT: optical coherence tomography; SWAF: Short-wavelength fundus autofluorescence; ERM: epiretinal membrane; iHRF: intraretinal hyper-reflective foci; EZ: ellipsoid zone; RPE: retinal pigment epithelium, OD: Oculus Dexter for right eye, OS: Oculus Sinister for left eye, OU Oculus Uterque for both eyes; LP Light Perception.

Table S4. List of primers used for *RPGR*^{ORF15} exon 15 Sanger sequencing.

Type	ID primer	Primer (FASTA)	Amplicon dimension (base pair)
Primers for PCR	ORF15_F3	5'-GACTAAACCCATAATATCCAAATCCA-3'	1953
	ORF15_R6	3'-GCCAAAATTTACCAGTGCCTCCTAT-5'	
Primers for Sequencing	ORF15_R7bSeq	3'-CCTTCCTCCTCTTCCCCCTCA-5'	N/A
	ORF15_R8bSeq	3'-TCCTTCCTCCTCTTCCCCCTCCCA-5'	
	ORF15_R9Seq	3'-CCCTGTGTGTTAGTAACTGAC-5'	
	ORF15_R5Seq	3'-ACTGGCCATAATCGGGTCACAT-5'	
	ORF15_R4Seq	3'-CTCTCCTTCCTCCTTTTCAC-5'	
	ORF15_R11Seq	3'-CAATTTAATAACACGTAATGAGTG-5'	

Table S5. Best corrected visual acuity (BCVA) data from both eyes of the patients with *RPGR*-related cone and cone-rod dystrophies. All data are presented as mean ± standard deviation. CI: confidence interval.

	Number of observations	Right Eye	Left Eye	Mean absolute difference	(95% CI interval) Lower	Upper	P value*
BCVA at last visit, logMAR	33	1.12 ± 0.84	1.04 ± 0.77	0.17	-0.36	0.70	0.35
Annual rate of BCVA decline, LogMAR/y	26	0.04 ± 0.06	0.03 ± 0.06	0.073	-0.02	0.04	0.80
Slope		0.04 ± 0.08	0.03 ± 0.06	0.045	-0.09	0.18	0.53
Intercept		-0.90 ± 3.34	-0.54 ± 3.14	1.89	-3.82	7.59	0.46
Annual rate of central hyperAF ring enlargement	19						
Horizontal diameter, µm/y		42.88 ± 48.98	45.71 ± 47.13	3.46	-28.8	34.46	0.75
Slope		43.16 ± 47.81	44.04 ± 41.78	12.12	-28.66	30.42	0.52
Intercept		228.60 ± 1689.24	212.78 ± 1479.13	157.88	-1028.86	1060.5	0.09
Vertical diameter, µm/y		39.44 ± 40.90	38.12 ± 41.54	1.46	-25.80	28.44	0.67
Slope		39.36 ± 40.57	38.33 ± 39.46	16.78	-25.3	27.36	0.27
Intercept		-24.16 ± 1658.86	-21.10 ± 1432.58	29.78	-1016.74	1022.86	0.08
CRT at last visit, µm	35	148.97 ± 27.17	148 ± 26.17	3.8	-11.75	13.69	0.18

*Wilcoxon signed-rank test; CRT: central retinal thickness
